# Remarks on the Internal Use of Cantharides in Gleet and Leucorrhœa, Illustrated by Cases

**Published:** 1806-05

**Authors:** John Roberton

**Affiliations:** Surgeon, Edinburgh; and Member of the Royal Medical Society


Remarks *on the* Internal Use *of* Cantharides *in* Gleet *and* Leucorrhœa, *illustrated by* Cases. *By* John Roberton, *Surgeon, Edinburgh; and Member of the Royal Medical Society.*

MUCH dispute, controversy and error would be prevented in medicine, as well as in the other departments of science, if ideas were expressed with more perspicuity of language, and the subjects of inquiry described with more precision than is usually done. The truth of this remark is well exemplified in what authors have said concerning the medical properties of Cantharides, which have been with equal ardour condemned as the most pernicious, or praised as the most salutary of substances, and, perhaps, with equal judgment also; since the one approved without understanding in what circumstance, or why it was useful; and the other rejected, without inquiring why it had been unsuccessful. This, like every other most powerful agent, when injudiciously or rashly employed, must have very pernicious effects, but when rationally and deliberately, its effects are not less conspicuously salutary.

In the consideration of every inflammatory disease, it is of the utmost importance to distinguish accurately between inflammation properly so called, and its consequences, wherefore we venture thus to distinguish gonorrhœa and gleet, or blenorrhagia.

Gonorrhœa, a contagious disease, distinguished by an active inflammation affecting the urethra, producing a discharge of puriform contagious matter from that passage.

In gleet, there is an atony of the urethra, the effects of active inflammation or other debilitating causes, accompanied with a glary or ichorous discharge, which is not contagious.

It seemed unnecessary to express these definitions in such a manner as to suit both sexes, since the mind can easily make allowance for the difference of structure, and perceive that the facts signified are equally applicable to both.

These two conditions, in principle and in practice, are completely analogous to the states of active and passive inflammation, as distinguished by the most correct and systematic writers; nor is there any rule of medical practice more firmly established, than that in the first, strong stimulants are as hurtful as they are beneficial in the second.

Accordingly, we need not be surprised that one person using the most active substance of cantharides in the gonorrhœa, as above defined, should meet with chagrin and disappointment, and condemn it in gonorrhœa; while another, who used it in gleet, which was also named gonorrhœa, should, as we do, from undoubted experience of its superior efficacy, recommend it to the notice of practitioners as a most valuable addition to the resources of the healing art.

We might have suspected from the relation of the contradictory effects, which this substance is described to have produced, what was the source of the error; but if this suspicion be corroborated by the facts contained in the writings of those who have prescribed it, the gonorrhœa has been confounded both in name and and in practice with gleet. If, where cantharides have been unsuccessful in removing discharges from the urethra, these have been gonorrhœas, but where successful, gleets; in fine, if positive experiment prove, that by inducing gonorrhœa, it cures gleet; will not this point be established, that gonorrhœa being confounded with gleet, is the cause of the opposite sentiments of writers with regard to the effects of cantharides in certain discharges from the urethra?

In the same manner we may, I presume, reconcile the difference of opinion with regard to the use of the corrosive muriate of mercury in gonorrhœa.

That the word gonorrhœa was employed to signify the discharge from the urethra in both the conditions above pointed out, the slightest attention to authors will amply satisfy any one; indeed, the word etymologically denotes a flow of semen, and has been applied indiscriminately to all discharges from the urethra; it is only in the advancement of science that the necessity of words precisely defined becomes urgent.

Dr. Greenfield * relates many cases of gonorrhœa, which he and others cured by means of cantharides; but if we examine the histories of the affection which they treated, we shall be convinced that they are those of gleet: and the phrases, violent gonorrhœa, virulent, painful, &c. which other writers employ to designate the affections in which the cantharides were unsuccessful or hurtful, show that these were discharges accompanied with active inflammation, or real gonorrhœas.

Lastly, I have not only the testimony of authors, but have found by indubitable experiment, that cantharides induce active inflammation with puriform discharge before gleet is remedied; how then could authors but fail in their attempts to remove active inflammation using a most inflammatory substance?

On examination of the facts, we firmly believe that those pernicious effects which have succeeded the use of cantharides, and which excited such horror and aversion to its exhibition in disease, have, as in the instance of gleet and gonorrhœa, principally originated in the want of discrimination between inflammatory action and its consequences.

Previously

* On the internal use of cantharides.

Previously to my employing cantharides in gleet, I had not consulted authors on the subject, nor am I ashamed to confess it, since the life-time of any individual would scarcely be sufficient simply to read over, far less to examine, the merits and doctrines of one twentieth part of the books written on the powers of particular medicines; besides, when we consider how useless the most of them are, a man has not much reason to regret, that he is not endowed with dull perseverance enough to pore over them.

Having since, however, examined the works of authors on this subject, I am happy to find that the experience of some of the most eminent medical men has found cantharides as efficacious a remedy for gleet as I have done; among these I may mention Bartholine, Hoffman, Lister, &c.

When I first employed cantharides in gleet, I only knew that this substance was a powerful stimulant taken internally, and produced painful effects on the urinary organs; and as pain generally indicates a certain degree of inflammation, I thought, that in inveterate gleets, where there is both general debility and exhaustion, with torpor and atony in the parts of generation, this substance might have considerable influence in restoring both the general and local activity; and the experiment, when made, exceeded in success my utmost hopes.

In order to demonstrate the truth of this assertion, I shall relate one case selected out of many; averring, that when exhibited with due caution, the cantharides effect the cure without vomiting, strangury, or any of those alarming symptoms, which we have been taught to dread, as the inevitable consequences of its administration.

S———d, aged 55, a small meagre man, on the 20th of April, 1804, mentioned to me that he laboured under an uncommonly severe gleet, at the same time saying, that he had no hopes of cure, for he had tried all the common prescriptions without advantage; but as the effects of the disease were now very severe, he was willing to submit to any thing likely to afford him relief; as, to use his own phrase, "life was become a burthen to him." I inquired minutely into his case, and found, that he had been affected with a discharge of this nature from the urethra, for about twenty years, which he attributed to the effects of a bad habit, originally commenced at school, and since aggravated by repeated claps.

Such was his situation, that besides a continual discharge from the yard, emission succeeded the most trifling erection, erection, and straining at stool had the same effect, which was followed by a great languor and depression of spirits.

Though married, connubial enjoyment was beyond his faculties; but this was not his only misfortune, head-ache, loss of appetite, lumbago, incontinence of urine; in fine, general emaciation and debility threatened the speedy termination of his life.

All the most common means had been already employed to remove his complaint; accordingly, to make him undergo a repetition of the same, was neither consonant to my feelings, nor suitable to the state of my patient, I therefore prescribed as follows:

℞. Tinct. Cantharid. ʒiß. Sp. Lavend. comp. ℈j. Aq. font. ℥vij. M. Cap. coch. mane et vesp.

This quantity having no effect, the dose was increased on the 23d to ʒij.; this likewise had no effect, and on the 26th was augmented to ʒiiß. which, though taken in the course of two days, an increased dose was still necessary. On the 28th, ℥ß. was prescribed with the above quantity of water, yet ineffectual; and this being all taken, the quantity was on the 30th increased to ℥j. of the tincture, which was taken in four days, and at this ratio he continued to the 8th of May. Between the 8th and 10th, he took half an ounce per diem; but inflammatory symptoms of the urinary organs appearing, for the first time, the dose was diminished to ʒij. in the day, and so on to the 17th; and whenever the signs of inflammation commenced, the matter of the gleet assumed the appearance, in some measure, of laudable pus, accompanied with chordée and severe erections, but yet no degree of stranguary; but what was somewhat remarkable, the erections were not now followed by those emissions, languor, sadness, and sudden impotence, which had been previously so certain consequences of that state.

The discharge from the urethra having now the form of the matter of a most inflammatory gonorrhœa, it was deemed unnecessary to persist in the use of the remedy, and unequivocal inflammation being indicated, it seemed prudent on the 18th to employ an injection, for the purpose of mitigating the symptoms; accordingly, a scruple of the sulphate of copper dissolved in six ounces of water, was ordered to be used three or four times a day, and, contrary to my expectation, the cure seemed to be complete in the course of four days from this date.

He left Edinburgh soon afterwards, and repaired to London, where his wife was, and in a short time he had an an increase of his family. He remained perfectly well in January, 1806.

Having for several years found the cantharides a very useful remedy in gleet, I became imperceptibly acquainted with the phenomena which succeeded its admission into the system; I had uniformly observed that it rendered the mind more cheerful, strengthened the circulation, promoted the animal functions, appetites, and evacuations, and greatly mitigated that sense of general debility, as well as of pain and feebleness in the loins and joints, of which, those who have long laboured under this affection so much complain: these are the first effects; but in a space of time, longer or shorter, according to the state of the patient, the discharge begins to be inspissated, pain attacks the urethra, erections are frequent, and even accompanied with some degree of chordée; but these erections are not as formerly, of short duration, succeeded by languor, debility, depression of spirits, and spontaneous evacuation of a semen like fluid, but precisely resemble the erections in gonorrhœa, and the matter discharged is puriform, and often, after erection, mixed with semen, particularly during the night.

While my mind was impressed with these facts, some obstinate and very inveterate cases of leucorrhœa came under my care; and reflecting that the constitutional symptoms of leucorrhœa are precisely similar to those of gleet, the matter of the same appearance and consistence; and as the gleety discharge evidently depends on a debilitated state of the mucous membrane of the urethra, so does the leucorrhœal on that of the mucous membrane of the vagina; in short, that all the circumstances were completely similar; it appeared to me very probable, that cantharides might also remove leucorrhœa by invigorating the general system, and producing a change in the action of the mucous membrane concerned, as happened in gleet, and the event confirmed my opinion.

Mrs. L——n, aged 25, a little woman, mother of five children; the first lived only a few hours after birth; the three following were still born; and the last lived only about half an hour. Her general appearance indicated languor and debility; her eyes peculiarly dull and heavy; pulse feeble. On the 9th of October, 1805, I learned that she had laboured under fluor albus for five years and a half, and the attack commenced about two months before the birth of her first child.

She has, during that time, employed those remedies which which are usually prescribed in this disease; but notwithstanding, her complaint had gradually increased in violence, and was at length intolerable. The opinion of the medical men she had consulted, had induced her to believe that a complete cure was scarcely to be expected. Her pain of back was excruciating; her appetite impaired; and so far from being able to walk, she could not even stand or sit, without the utmost uneasiness. The discharge per vaginam too, was so copious and incessant, that, though she used cloths, &c. she was always afraid of being in company, lest marks on the floor should discover her condition; in addition to all which, she was frequently attacked by paroxysms of hysteria.

The discharge was of a glairy appearance and consistence. During the whole time of the affection, the menstruation has been regular and natural; and what is worthy of remark, the fluor albus evinced itself much worse during pregnancy than at other times, showing, as I would venture to allege, that the particular state of the mucous membrane of the vagina alone has no small share in promoting the disease.

The patient says, the flow was not in uniform quantity, but that an unusally great discharge was always preceded and announced by an excruciating pain in the region of the kidnies, on which occasion she always had the distinct sensation of something flowing, as it were, downwards from the loins, and the cloths applied to the parts were found wet with the discharge.

Is there not reason to conjecture that the mucous membrane of the urethra is equally affected with that of the vagina?

As in other cases I had been accustomed to use the tincture of cantharides with great success, I prescribed as follows:

℞. Tinct. Cantharid. ʒiiſs. Aq. font ℥vj. M. One table spoonful to be taken thrice a day.

October 12. Cantharides mixture repeated with ʒjv. and aq. font. ℥vj. and again repeated with the same quantity of cantharides on the 16th.

17th. She felt slight pain in the uterine region, extending down the vagina, particularly on making any exertion. The discharge was now somewhat thicker and whitish.

19th. Cantharides mixture repeated; in the evening the pain became very troublesome, even when sitting, and the discharge was much thicker and whiter. I now prescribed as an injection:

℞. Sulph.

℞. Sulph. Cupri. ʒß. Aq. font. ℥vi. M. To be thrown into the vagina thrice a day.

20th. Discharge and pain almost gone; when I desired her to take two tea spoons full of the cantharides mixture every three hours, till the pain should return: but before she had taken a sufficient quantity to produce this effect, the discharge resumed almost its former violence; cantharides increased to the quantity prescribed on the 16th.

21st. Discharge considerably diminished, attended with slight pain.

22d. No pain, although she has taken within the last twenty-four hours ʒiv. of the tinct. cantharid. The discharge now less, by more than two-thirds, than it was previously to the exhibition of the cantharides. I now ordered the injection to be omitted, and the following increased dose of the tinct. cantharides to be used.

℞. Tinct. Cantharid. ℥j. Aq. font. ℥vj. M. To be used as the former mixture.

23d. After taking one dose of the last mixture, she was for the first time sensible of pain in voiding urine. This was the period for the return of the menses, which accordingly appeared, but she was at the same time seized with a fever, and species of cynanche tonsilaris, that had raged epidemically in Edinburgh for some weeks. Tinct. cantharid. and injection now intermitted, as he fever, &c. continued to the first of November.

Nov. 2d. Fever gone; throat getting better after one of the tumors had suppurated and burst.

Menstruation still continuing, and of an alarming quantity, prescribed this day,

℞. Acidi Nitrici ʒij. Aq. font. ℔ij. M. One wine glassful thrice a day, till the flooding ceases.

7th. Flooding ceased; the discharge of the fluor albus considerably abated.

9th. Discharge much aggravated. General stimulants were now prescribed, because the system was much reduced by the fever, &c.

12th. Discharge much diminished,

13th. Discharge entirely gone; the general health much improved.

17th. Discharge returned in a slight degree, and of the same appearance and consistence as before the use of the cantharides.

21st. After earnest persuasion, on my part, she was prevailed on to recommence the tinct. cantharid. in small doses.

22d.

22d. Great sickness during the night; and inflammatory symptoms, with a puriform color in the discharge, came on before morning.

℞. Camphor, G. Mimosæ Niloticæ, aa. ℈j. Alcohol. q. s. ft. Massa in pilul. xv. dividend.

One of these pills was taken an hour after each dose of the cantharides mixture, which was to be taken thrice a day.

23d. Cantharides continued; very great pain this day; but as it has never become so severe as on the 21st, she has used none of the camphor pills.

24th. Discharge entirely gone. Cantharid. continued in smaller doses, but sufficient to maintain the suppurative action.

25th. Pain of head.

℞. Supertart. potass. ℥j. solve in jusc. bov. ℔j. To be taken presently; cantharid. continued; no return of the discharge.

26th. ℞. Tinct. Cantharid. ℈v. Aq. font. ℥vj. M. To be taken as formerly.

On the evening of this day, after having fatigued herself by walking, and having also neglected to take the cantharides since morning, the pain in the uterine region abated, and the discharge returned in a slight degree.

27th. Cantharides to be taken during this day, till the pain returns; pain and considerable difficulty in making water came on this night at bed time, with nearly a complete cessation of the discharge.

28th. Cantharides continued, and one of the camphor pills to be taken; discharge slight.

28th. Cantharides continued; discharge not entirely gone, but nearly so.

30th. Discharge entirely gone; cantharides continued, and the quantity last prescribed, repeated; considerable uneasiness, or rather pain in making water. Partial sickness this day and yesterday, with head-ache; supertart. potass. as above, which relieved these symptoms.

December 1st. Very early this morning there was an almost imperceptible discharge; cantharides and injection continued.

2d. Uneasiness extending along the vagina and uterine region, still kept up by the use of the cantharides; injected two or three times since yesterday, but no return of the discharge,

3d. The pain in the region of the kidnies, which heretofore invariably indicated the returning discharge, was felt this morning, but not followed by the discharge, or even the the smallest appearance of matter, per vaginam; in other respects as yesterday.

The menstruation not having returned at the regular period, there is reason to suspect the intervention of pregnancy; I have therefore ordered the dose of the cantharides to be diminished, but the injection into the vagina to be continued.

4th. Had a violent attack of hysteria this morning, occasioned by mental agitation. Such an attack never happened before, during the complaint, without its being succeeded by an uncommonly copious discharge, of which there is no appearance. Medicines to be continued as last prescribed.

5th. No discharge; uneasiness of the parts gradually abating.

6th. A repetition of the hysteric paroxysm from a similar cause.

7th. Early this morning the discharge reappeared, of a puriform nature, and continued to augment all this day; pain from the cantharides almost gone. Cantharides to be taken in increased doses, and the injection to be assiduously repeated.

8th. Discharge gone; pain of the parts returned; remedies as last directed, but in diminished doses.

10th. No discharge; pain gone; general health much better than she thinks it ever was since she laboured under this affection; is not oppressed with that despondency from which she felt very disagreeably in her former pregnancies.

14th. No cantharides taken since the 10th; pain and discharge gone; complains of sickness in the morning; loss of appetite; formerly when pregnant, she had none of the usually concomitant symptoms of that state, except the cessation of the menses, and the increase of size, at which time too, the discharge per vaginam was signally augmented.

20th. Previously to this date she had used the injection very irregularly. During the afternoon of yesterday, she had been walking, sailing, and dancing a good deal; suddenly awoke this morning with violent palpitation of heart, and agitation of mind, from a dream; slight return of the discharge; injection to be used more regularly; cantharides to be repeated in small doses.

22d. Vomits the cantharides immediately on taking it, and disgusted with it. Her common food is loathsome to her, and excites severe sickness and vomiting, but no pain in in the stomach or chest. The same occurs on getting up in the morning; same discharge, though scarcely perceptible. Cantharides discontinued on account of pregnancy; injection used once a day.

January 1st, 1806. Discharge has continued as on the 22d ult. She danced and fatigued herself a good deal this evening, but this was succeeded by no augmentation of the discharge, as on the former occasion, when she had been sailing and dancing. Could the cold sea-air produce any difference in the result? for on the first of January she fatigued herself much more than on the former occasion.

5th. Says the discharge is still perceptible. I asked her what it was, compared with what it had been? She replied emphatically, "It is not the ten thousandth part of what it formerly was; and I feel myself as well in my general health as ever I was in my life, except that my bones and joints are still sore from the exertion of the first day of this year."

The above case was selected, because it indicated the greatest variety of circumstances; but there are others, which shall afterwards be detailed, of equal duration and obstinacy, which have been completely cured without relapse.

I may mention that cantharides seem formerly to have been used successfully in leucorrhœa, though the nature of the affection which this medicine removed, does not seem to have been understood by those who employed it. Dr. Greenfield, for example, had adopted a notion, that almost all such discharges as those of gleet and leucorrhœa depended on ulceration of the bladder, and that cantharides were an infallible remedy for such ulcers, just as in our own times some promulgate, that almost all affections of the organs of generation, nay, even of the head, stomach, bowels, &c. depend on strictures of the urethra, for which bougies and caustics are the only specifics.

The following case, communicated to Dr. Greenfield by Charles Bernard, Surgeon to Queen Anne, was evidently one of leucorrhœa mistaken for ulceration of the bladder.

"A certain lady of quality, who for a long time had been afflicted with an ulcer in her bladder, and had applied herself to many of the College of Physicians in London, but without any manner of relief; she mightily implored, from time to time, my assistance, which I as often denied, because the distemper was both difficult of cure, and chronick withal, and the patient daily voided an incredible quantity of mere fœtid pus; at last I was prevailed upon to undertake, or rather attempt the cure, and the more so, that I might have the opportunity of trying the method so mightily recommended by Dr. Greenfield, viz. the internal use of cantharides in curing ulcers in the bladder. I began the trial by two grains of cantharides, to be taken twice a day, from the 26th of March to the 2d of April, but by the use of these a dysuria ensued, wherefore we omitted them till 24th April, and prescribed balsamics in their room; but what was very specially observable was, that the quantity of purulent matter (which she used daily to evacuate) was wonderfully diminished.

"From the 24th till the 26th of April, was repeated two grains of cantharides twice a day as before.

"From the 26th of April till the 1st of May, we advanced to three grains of cantharides, intermingling balsamics by intervals; meanwhile a strangury continued to be very troublesome, but the purulent matter lessened daily, and at length wholly disappeared."

Having collected a much greater number of facts and observations than could be admitted into a periodical publication, or than my other affairs would allow me immediately to prepare for the press, I intend still further to prosecute the present inquiry, and during the intervals of professional employment, to prepare for publication the result of my future experience on this subject.

*Edinburgh*, 1806.

